# Electrically stimulable indium tin oxide plate for long-term in vitro cardiomyocyte culture

**DOI:** 10.1186/s40824-020-00189-0

**Published:** 2020-05-27

**Authors:** Sung-Hwan Moon, Young-Woo Cho, Hye-Eun Shim, Jae-Hak Choi, Chan-Hee Jung, In-Tae Hwang, Sun-Woong Kang

**Affiliations:** 1 Department of Medical Science, School of Medicine, Konkuk University, Seoul, South Korea; 2 Drug Safety and Toxicity Evaluation Team, New Drug Development Center, Osong Medical Innovation Foundation, Cheongju-Si, Chungbuk South Korea; 3 Research Group for Biomimetic Advanced Technology, Korea Institute of Toxicology, Daejeon, South Korea; 4 Department of Polymer Science and Engineering, Chungnam National University, Daejeon, South Korea; 5 Research Division for Industry and Environment, Advanced Radiation Technology Institute, Korea Atomic Energy Research Institute, Jeonbuk, South Korea; 6 Department of Human and Environmental Toxicology, University of Science and Technology, Daejeon, South Korea

**Keywords:** Indium tin oxide, Electrical stimulation, Neonatal rat ventricular myocytes, Stem cells

## Abstract

**Background:**

We investigated whether electrical stimulation via indium tin oxide (ITO) could enhance the in vitro culture of neonatal rat ventricular myocytes (NRVMs), which are important in vitro models for studying the mechanisms underlying many aspects of cardiology.

**Methods:**

Cardiomyocytes were obtained from 1-day-old neonatal rat heart ventricles. To evaluate function of NRVMs cultured on ITO with electrical stimulation, the cell viability, change of cell morphology, immunochemistry using cardiac-specific antibodies, and gene expression were tested.

**Results:**

Defined sarcomeric structure, cell enlargement, and increased distribution of NRVMs appeared in the presence of electrical stimulation. These characteristics were absent in NRVMs cultured under standard culture conditions. In addition, the expression levels of cardiomyocyte-specific and ion channel markers were higher in NRVMs seeded on ITO-coated dishes than in the control group at 14 days after seeding. ITO-coated dishes could effectively provide electrical cues to support the in vitro culture of NRVMs.

**Conclusions:**

These results provide supporting evidence that electrical stimulation via ITO can be effectively used to maintain culture and enhance function of cardiomyocytes in vitro.

## Background

Over decades, diverse types of cells have been cultured for various purposes, ranging from basic research to cell application development [1–3]. Many studies have been conducted to develop in vitro cell culture methods that can efficiently enhance and maintain functions of various types of cells [4, 5]. However, each cell type requires different culture conditions in vitro due to its unique and innate characteristics. Furthermore, terminally differentiated cell types, such as cardiomyocytes and neural cells, need to be functionally mature for therapeutic purposes. Therefore, the cell culture method must be sought for suits the characteristics of each cells.

Recently, cardiomyocytes used to treat heart failure can regenerate the lost muscle and to screen efficacy and toxicity of drugs that are in their initial stages of development [6–10]. However, cardiomyocytes hardly proliferate after birth but grow as individual cells to display functionally and structurally mature phenotypes [11, 12]. In addition, these cells have membranes which allow sodium, calcium, potassium ions to slowly move inward and out the cell for systole and diastole [10]. To support these characteristics in vitro, appropriate conditions are required to mimic their natural niche environment [13]. Mimicking the natural environment is one of the methods used to enhance the function for culture of cardiomyocytes in vitro. Indeed, exogenous electrical stimulation and physical surface patterns can induce the functional maturation of human pluripotent stem cell (hPSC)-derived cardiomyocytes [14, 15]. Based on these findings, modulating electrical stimulation is becoming an increasingly attractive biomimetic approach to provide an ideal in vitro culture setting to support fully functional cardiomyocytes. This method often requires conductive materials for provision of electrical stimulation.

Recently, indium tin oxide (ITO) and graphene, cell culture substrates that are more conductive than conventional plastic cell culture dishes, have been utilized for in vitro culture [16, 17]. Thin films of ITO are ideal for cell culture due to their high optical transparency and stability under warm and humid conditions along with their nontoxicity and excellent electrical conductivity [18]. Indeed, various cell types have been cultured on ITO-coated surfaces without any overt disadvantages or detrimental consequences [19]. However, whether ITO-coated surfaces are suitable for the in vitro culture of neonatal rat ventricular myocytes (NRVMs) is currently unclear. Therefore, the objective of this study was to use ITO-coated dishes as conductive culture substrates to examine the effect of electrical stimulation on the in vitro culture of NRVMs.

## Methods

### Materials

ITO-coated glass slides (75 × 25 mm) were obtained from Sigma (St. Louis, MO, USA). These ITO-coated glass slides had surface resistivity of 8–12 Ω with nominal transmittance > 83% and thickness of 1.1 mm. The ITO was washed by sonication (15 min each) using acetone, ethanol, and 2-propanol (Sigma).

### NRVM isolation and electrical stimulation

Cardiomyocytes were obtained from 1-day-old neonatal rat heart ventricles. Sprague Dawley rats were used following a protocol approved by the Institutional Animal Care and Use Committee of the Korea Institute of Toxicology (IACUC 17-1-0190) and the Guidelines for the Care and Use of Laboratory Animals of the National Research Council. The ventricle was minced in 0.2% solution of trypsin in Hank’s balanced salt solution (HBSS, Thermo Scientific, Grand Island, NY, USA) and then subjected to a series of digestions (15 min, 37 °C, 500 x g) with a mixture of 0.05% collagen type II and 0.06% pancreatin in HBSS (enzyme solution). The first digest was discarded. The cell suspension from the 4th digestion was centrifuged (330 x g, 10 min) and then washed with HBSS for resuspension. Subsequently, the cells were resuspended in high glucose DMEM (Gibco BRL, Gaithersburg, MD, USA) containing 15% fetal bovine serum (FBS, Gibco) and then plated onto a 100-mm cell culture dish at 37 °C for 2 h. The cells remaining in suspension were collected for further culture on ITO. Before seeding with cells, the ITO slides were coated with 2% gelatin for 30 min. Cell number and viability were determined with a hemocytometer and trypan blue staining, respectively. After cardiomyocyte seeding (5 × 10^4^ cells/well) on ITO slides, stimulation was applied at 5 V with a pulse duration of 10 ms at a pacing frequency of 2 Hz for 21 days. The cells were maintained in a defined medium. The control cells received no stimulation. During the experimental period, the medium was replaced with fresh DMEM supplemented with 10% FCS (Gibco) every 2 days.

### Cell viability assay

Cell viability was analyzed using a LIVE/DEAD Viability Assay Kit (Invitrogen, Grand Island, NY, USA). Briefly, cells in 5 mL of cardiomyocyte culture media were mixed with 1 μL of calcein AM solution (LIVE) and 5 μL of ethidium homodimer-1 solution (DEAD) followed by incubation at 37 °C for 40 min in a 5% CO_2_ incubator. NRVM staining was analyzed under a Ti-2000 fluorescence microscope (Nikon, Japan).

### Measurement of NRVM alignment

Cell morphology was assayed with phase contrast microscopy. Cultured plates were removed from the incubator on an inverted microscope (Eclipse TS100, Nikon). Live images were captured using a digital camera (U3, Nikon) and analyzed with NIS software (Nikon). Cell area and circularity were determined by tracing the outline of 100 individual cells from four different areas of the coverslip using ImageJ software and standard analysis plugins [8]. The average value was calculated (*n* = 30).

### Immunochemistry

Cells were fixed in 4% PFA at 4 °C for 15 min and permeabilized with 0.1% Triton X-100 in PBS (Welgene, South Korea) for 5 min. After treatment with blocking solution containing 5% normal goat serum for 30 min, cells were stained with primary cardiac-specific antibodies against sarcomeric α-actin (Sigma) and connexin43 (BD Biosciences, Bedford, MA) at 4 °C overnight. The cells were washed three times with PBS and then stained with TRITC-conjugated secondary antibodies (Molecular Probes Inc., Eugene, OR, USA) at room temperature for 1 hour. After washing with PBS, nuclei were stained with DAPI (Invitrogen). All images were analyzed using LSM 510 and 710 META confocal microscopes (Carl Zeiss Inc., Oberkochen, Germany).

### Gene expression

Total RNA was isolated from the cells cultured in the presence or absence of electrical stimuli using TRIzol reagent (Invitrogen) according to the manufacturer’s instructions and used for cDNA synthesis with the SuperScript II Reverse Transcription Kit (Invitrogen). Quantitative real-time PCR (qRT-PCR) analysis was performed using SYBR Green gene expression assays (Roche, Mannheim, Germany) with GAPDH as an internal control. The primer sequences used in qRT-PCR are shown in Supplementary Table 1. The gene expression level was calculated using the ΔΔCt method (*n* = 3).

### Statistical analysis

All data are presented as mean values ± standard deviations. Comparisons between two groups were performed using Student’s t-test. A *P* value of less than 0.05 was considered statistically significant. Data graphs were fit with GraphPad Prism version 7.

## Results

### Electrical stimulation of NRVMs on ITO-coated slides

**Fig. 1:**
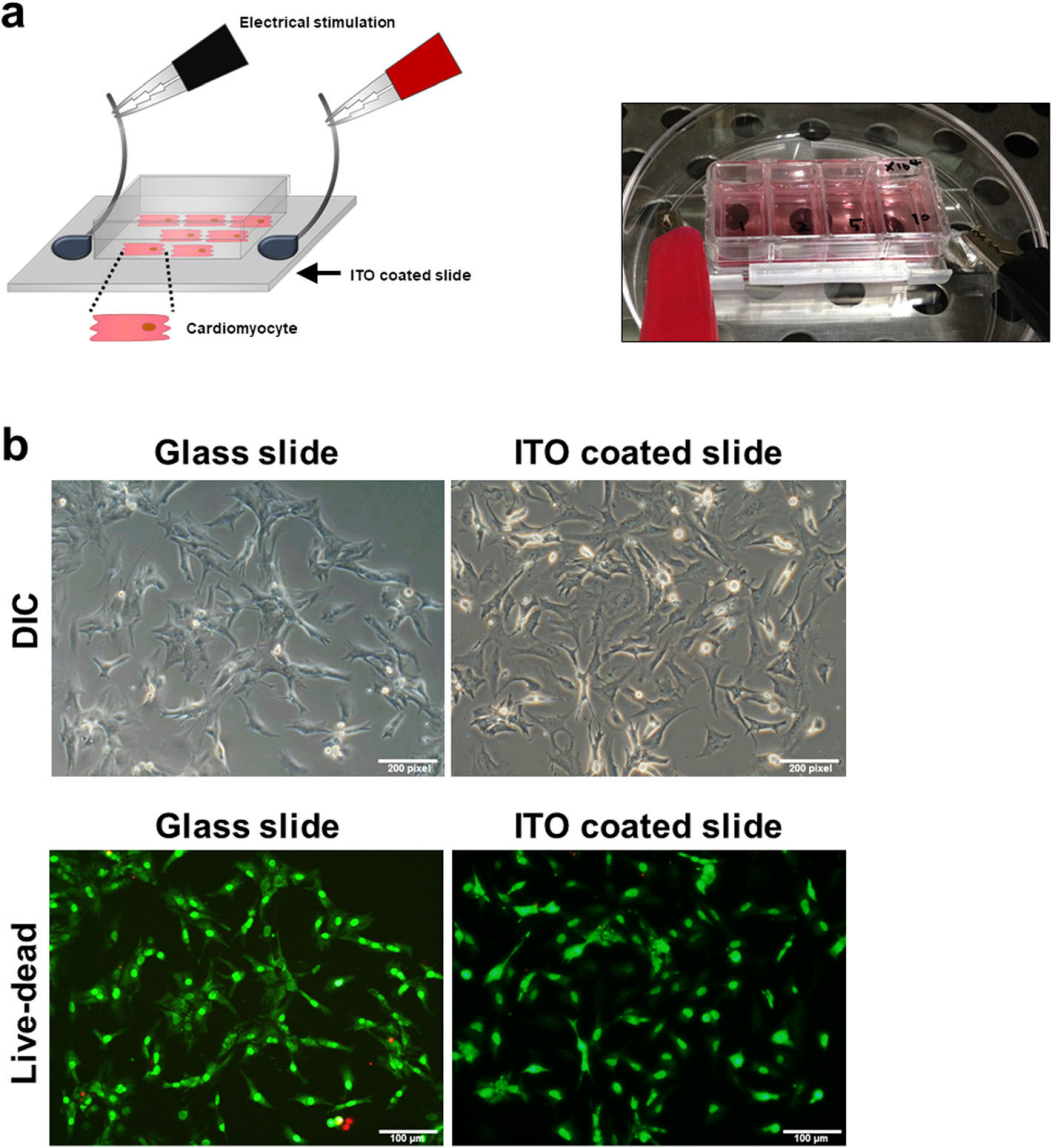
Electrical stimulation and morphology of NRVMs on ITO-coated dishes compared to the control. **a** Brief schematic showing the electrical stimulation of NRVMs on ITO-coated dishes. **b** Morphology and live-dead staining of NRVMs after seeding. Cells attached and survived on ITO-coated substrates. No significant differences were observed between the two groups

**Fig. 2:**
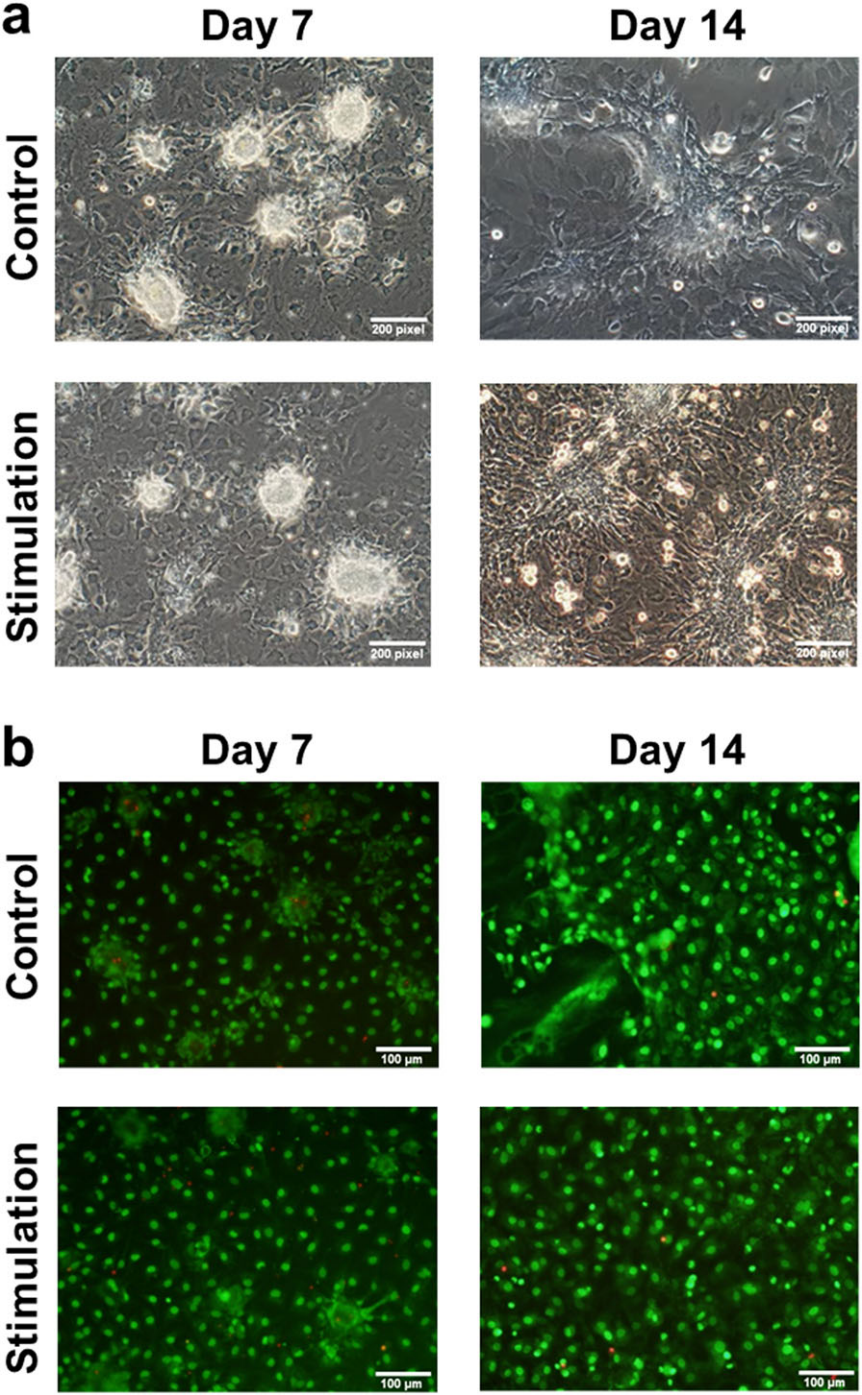
Long-term culture of cells on ITO with and without electrical stimulation. **a** Bright-field images of cells. **b** Live-dead staining images at day 7 and day 14 after culture

The overall scheme for the electrical stimulation of NRVMs on ITO-coated slides is illustrated in Fig. 1a. Briefly, a total of 5 × 10^4^ cells was seeded onto an uncoated glass slide and an ITO-coated slide and cultured in DMEM supplemented with 10% FCS for 3 days. Based on microscopic observations, cells attached on both slides within 24 h of seeding. There was no significant difference in attachment rate between the two groups (with and without ITO stimulation). In addition, attached cells on both slides remained viable based on a live-dead assay (green fluorescence, Fig. 1b), indicating that ITO-coated slides were suitable for our intended investigation. Next, we compared the growth characteristics of unstimulated NRVMs with those of NRVMs cultured in the presence of electrical stimulation. The culture conditions used for NRVMs in both groups were identical except that the NRVMs on the ITO-coated slides were cultured under continuous electrical stimulation with a monophasic pulse amplitude of 5 V, pulse duration of 10 ms, and stimulation frequency of 2 Hz. The overall changes in growth pattern during culture were very similar between groups. Cell aggregates appeared early in the culture period. Eventually, they all formed a monolayer of cells (Fig. 2a). In addition, cell viability was unaffected by the presence or absence of electrical stimulation (Fig. 2b). In general, most cells cultured on ITO-coated slides had higher beating rates than unstimulated NRVMs (Supplementary 2. live images a, b, c and d).

### Electrical stimulation enhances the sarcomere structure of NRVMs

**Fig. 3:**
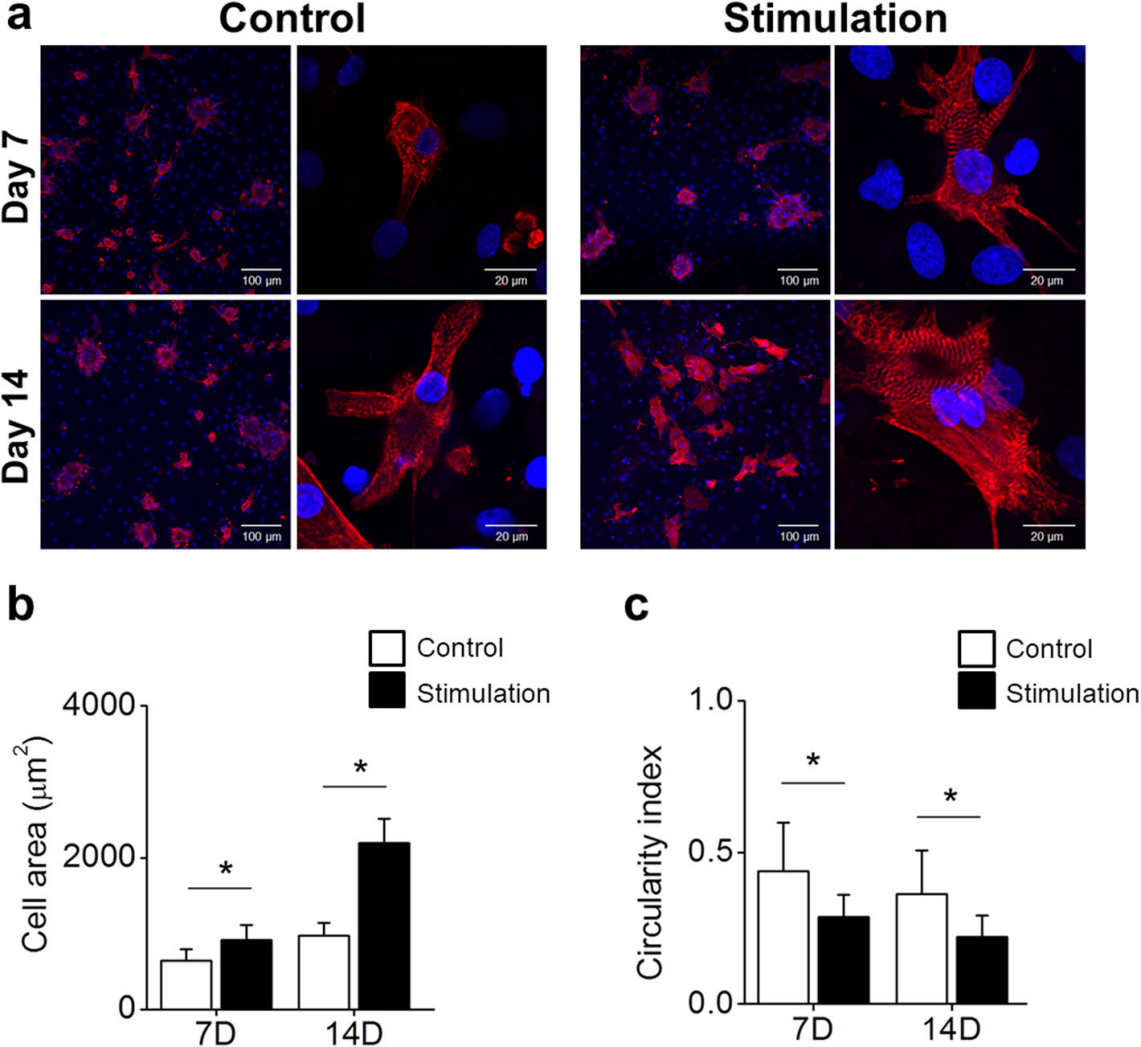
Structural and morphological changes in cells during culture. **a** Sarcomere α-actin (red) and nuclei (blue) were stained to observe structural changes. Calculation of (**b**) cell area and (**c**) circularity. Increased cell area and decreased circularity at day 14 might indicate natural cardiomyocyte development. *, *P* < 0.05

The sarcomere is the basic contractile unit of cardiomyocytes. To examine whether electrical stimulation affected the number and structure of sarcomeres in NRVMs, we performed immunostaining using an antibody against α-actinin at day 7 and day 14 after culturing. The differences in sarcomere structures between unstimulated and electrically stimulated NRVMs were hardly noticeable when they were examined at 7 days after culture. However, on day 14, NRVMs cultured in the presence of electrical stimulation developed well-organized sarcomere structures in enlarged cardiomyocytes compared to unstimulated cells (Fig. 3a). Since sarcomeres are responsible for the contractility of cardiac myocytes, this may explain the higher beating rate observed in this study (Supplementary 2. live images a, b, c and d). NRVMs cultured under both conditions showed a significant increase in cell area. However, the increase in cell area was higher in the stimulated group than in the control group (Fig. 3b). Furthermore, the morphology of electrically stimulated NRVMs became more elongated based on their significantly lower circularity index than that of unstimulated NRVMs (Fig. 3c). Overall, changes in these parameters indicative of NRVM functionality (sarcomere structure, cell size and elongated morphology) suggest that electrical stimulation could be used as an important mediator for better maintenance of NRVMs in vitro.

### Expression levels of specific cardiomyocyte markers and ion channels in electrically stimulated NRVMs

**Fig. 4:**
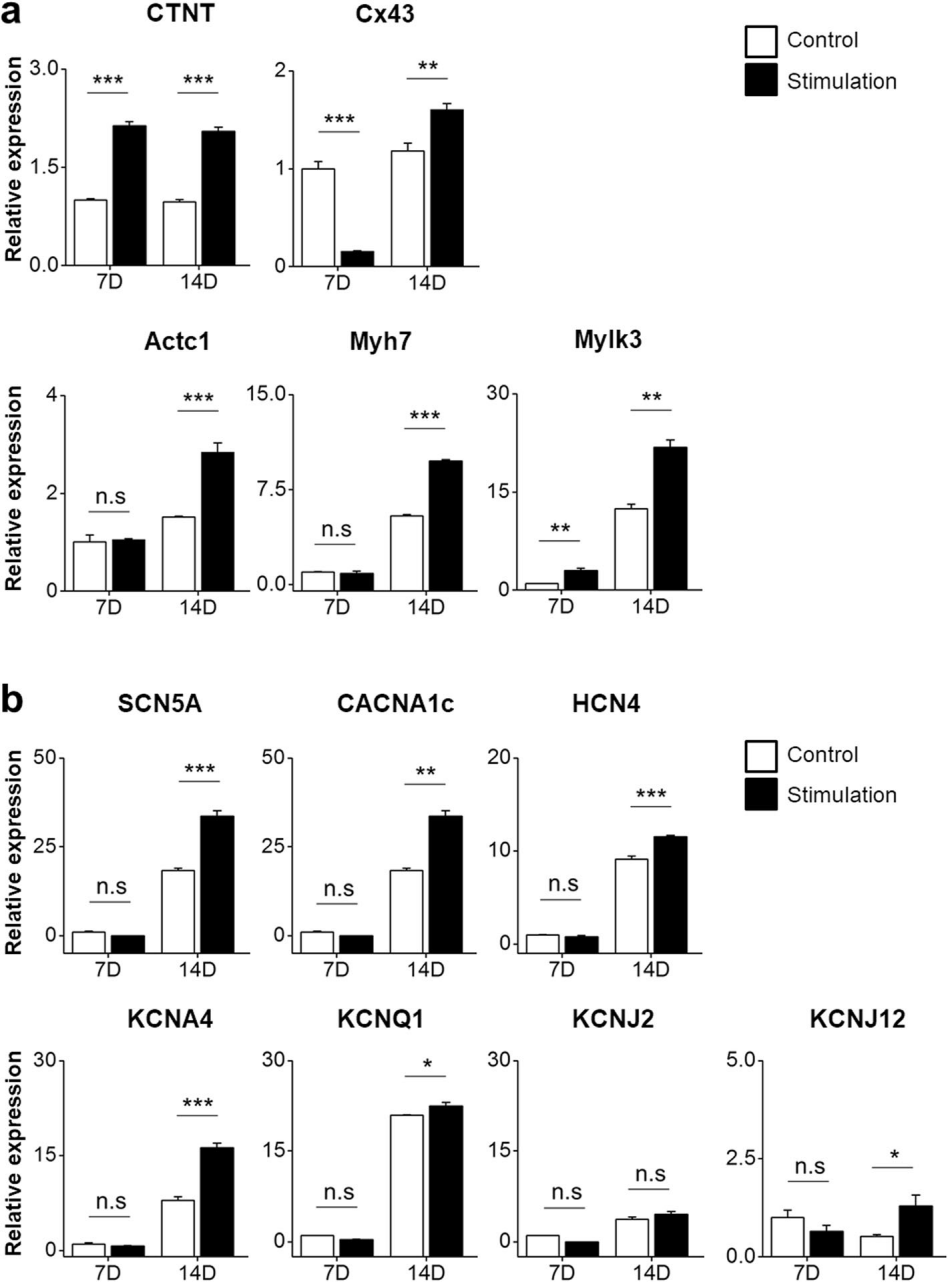
Relative expression of cardiomyocyte-specific genes and ion channel genes by qRT-PCR. **a** The expression levels of cardiomyocyte-specific genes in the electrical stimulation group were higher than those in the control group. **b** The expression levels of ion channel genes except KCNJ2 and KCNJ12 in the electrical stimulation group were significantly increased at day 14 compared to those in the control. *, *P* < 0.05; **, *P* < 0.01; ***, *P* < 0.001

To analyze the expression levels of cardiomyocyte-specific markers (cTnT, Cx43, Acta1, Myh1, and Mylk3) in both groups (control and electrical stimulation), qRT-PCR was performed. The expression levels of cardiomyocyte markers (cTnT, Cx43, Actc1, Myh7, and Mylk3) in the electrically stimulated group were higher than those in the control at day 14 (Fig. 4a). In particular, the expression level of connexin43 was significantly higher on day 14 than on day 7. Connexin43 is a marker for gap junctions [20], and an increase in its expression indicates functional enrichment and maturation through increased formation of gap junctions required for cell interaction and signal transmission. Next, we examined the expression levels of a sodium channel gene (SCN5A), calcium channel gene (CACNA1c), and potassium channel genes (HCN4, KCNJ2, KCNQ1, KCNJ2, and KCNJ12) (Fig. 4b). The expression levels of sodium, calcium, and potassium channel genes in the electrical stimulation group were significantly (*P* < 0.05) higher than those in the control group (Fig. 4b). Therefore, the ion channel genes in the control NRVMs had lower expression than those in the electrically stimulated NRVMs, implying that stronger cell-cell interactions due to increased gap junctions in the electrical stimulation group might have resulted in a natural increase in ion channels. Based on the observed cardiomyocyte-specific and ion channel gene expression profile, it is conceivable to conclude that ITO/electrical stimulation might exert favorable benefits to prolong the maintenance of functional cardiomyocytes.

## Discussion

Electrical signals are known to play an important role during cardiac development in embryonic tissue [21, 22]. In addition, previous studies have reported that exogenous electrical stimulation can enhance cardiomyogenic differentiation of stem cells [21, 23]. However, it remains unclear how electrical stimulation affects cardiomyocytes during culture period for maintenance of cardiomyocytes not cardiomyogenic differentiation in vitro. In this study, we examined the feasibility of using ITO as a biomimetic system to deliver electrical signals for favorable environment of cardiomyocytes in vitro. The principal findings of this study are that electrical stimulation results in well-organized sarcomere structures and significant upregulation of cardiac-specific gene expression.

The NRVMs provide a useful tool in cardiac research because they are relatively easy to isolate compared to their counterparts derived from adult hearts [24]. However, a multitude of in vivo environmental and cellular cues that govern their functions are absent from in vitro settings. An increasing body of evidence has suggested that exogenous electrical stimuli have beneficial effects on in vitro cultured cardiomyocytes [25, 26]. In this study, ITO has been used for electrical stimulation setting. The NRVMs showed no differences in terms of cell attachment and viability on ITO surfaces compared to glass slides, even in the presence of varying levels of electrical stimulation. However, the cells cultured on ITO-coated surfaces with electrical stimuli demonstrated a well-defined sMHC-positive sarcomeric structure compared to those cultured in the absence of electrical stimulation (Fig. 3a).

A significant increase in the expression of cardiomyocyte-specific markers and ion channel genes was also evident when electrical stimuli were applied to NRVMs (Fig. 4). The sustained higher expression level of cardiomyocyte-specific genes and ion channel genes suggest that, to a certain extent, electrical stimulation is a favorable condition for NRVMs in in vitro culture. Similar observations have been reported in other studies, showing that the electrical stimulation of human cardiomyocyte progenitor cells (CMPCs) using a custom-made stimulation-unit setup can increase the expression of a panel of CMPC-specific genes (MEF2A, GATA4, α-actinin, cTnl, Cx43, and SERCA2) compared to their expression in unstimulated counterpart cells [27]. In another study, electrical field stimulation via a microelectrode array (MEA)-compatible electrical stimulation platform increased the expression of connexin-43 (a gap junction marker) and contractile cardiac protein beta myosin heavy chain 7 (MYH-7) in neonatal rat cardiomyocytes (NRCs) in a 3-day experiment [28]. Electrical stimulation not only enhances key cardiomyocyte-specific gene markers during in vitro culture but also improves the morphology, differentiation, and functional maturation of cardiomyocytes [23, 29, 30]. Despite this influential role of electrical stimuli in cardiomyocyte development, the exact mechanism by which electrical stimuli induce their functional benefits remains largely elusive. One study has suggested that small amounts of reactive oxygen species (ROS) generated after electrical stimulation might elicit a cascade of signaling pathways involved in cardiac differentiation and development [31].

Elongation and alignment of cells in the direction of electrical stimulation are commonly observed after culturing cardiomyocytes in an electrical stimulation field [9]. This change in morphology was accompanied by improved contractility, conductivity, and formation of functional gap junctions. Such alignment was observed in our studies (Fig. 3), suggesting that complex interplays might exist between signaling pathways and other biological or mechanical preconditioning to drive the sustainability of functional cardiomyocytes upon electrical stimulation.

The use of in vitro cell cultures to predict in vivo effects is troubled with difficulties related to the loss their phenotype during culture. Culture of primary neonatal rat cardiomyocytes is limited by the loss of spontaneous contractile phenotype within weeks in culture [32]. This may be due to loss of contractile cardiomyocytes from the culture and that this is associated with CM dedifferentiation and cytoskeleton remodeling [33, 34]. In our study, culture method of NRVMs on ITO with electrical stimulation retained the expression of cardiomyocyte markers and displayed regular beating rate. These finding suggest that ITO with electrical stimulation can positively influence contractility of cardiomyocyte during culture and NRVMs cultured on ITO with electrical stimulation retain potential as test model for predictive drug screening in vitro.

## Conclusion

We demonstrated that ITO is a simple and viable biomimetic system to deliver electrical stimuli in order to create more favorable culture conditions of cardiomyocytes in vitro.

## Supplementary information

**Additional file 1: Supplementary 1. Table S1.** List of primers used in qRT-PCR

**Additional file 2: Supplementary 2. live images a, b, c, and d.** Comparison of live imaging between control at (a) day 7, (b) day 14 and stimulated groups at (c) day 7, (d). The beating area of the stimulated group gradually increased during culture compared to that in the control group.

**Additional file 3.**

## Data Availability

For data requests, please contact the authors.

## Abbreviations

ITO: Indium tin oxide
ROS: Reactive oxygen species
NRCs: Neonatal rat cardiomyocytes
MYH-7: Myosin heavy chain 7
MEA: Microelectrode array
CMPCs: Cardiomyocyte progenitor cells
NRVMs: Neonatal rat ventricular myocytes
hPSC: Human pluripotent stem cell
