# Supplementary figures and images for: Electrically stimulable indium tin oxide plate for long-term in vitro cardiomyocyte culture
Source: Biomater Res. 2020 May 27;24:10. doi: 10.1186/s40824-020-00189-0 (PMC7251917; doi:10.1186/s40824-020-00189-0)

**Supplementary 1. Table 1.** List of primers used in qRT-PCR


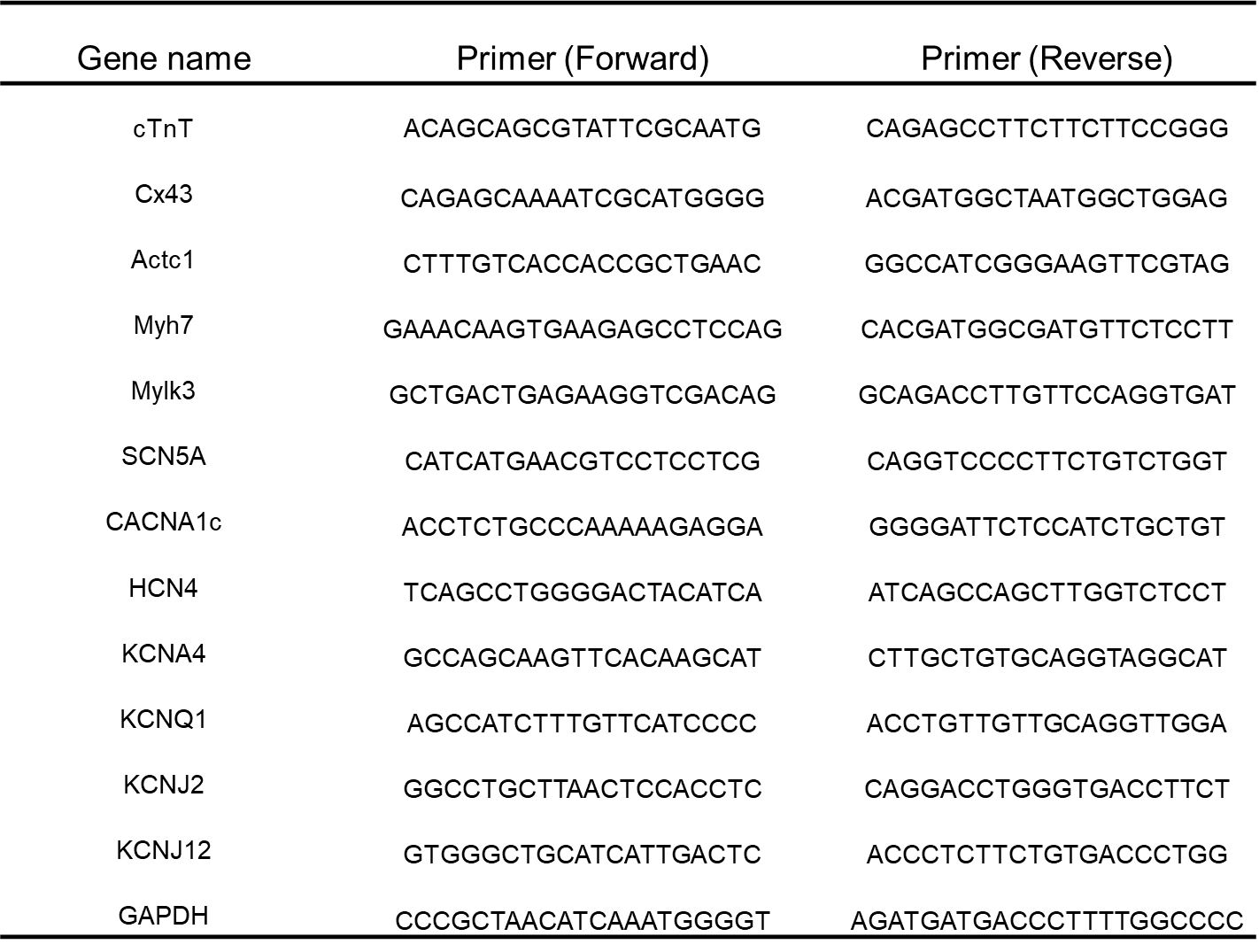

Supplement: Supplementary file 1 — Additional file 1: Supplementary 1. Table S1. List of primers used in qRT-PCR [file 40824_2020_189_MOESM1_ESM.docx]

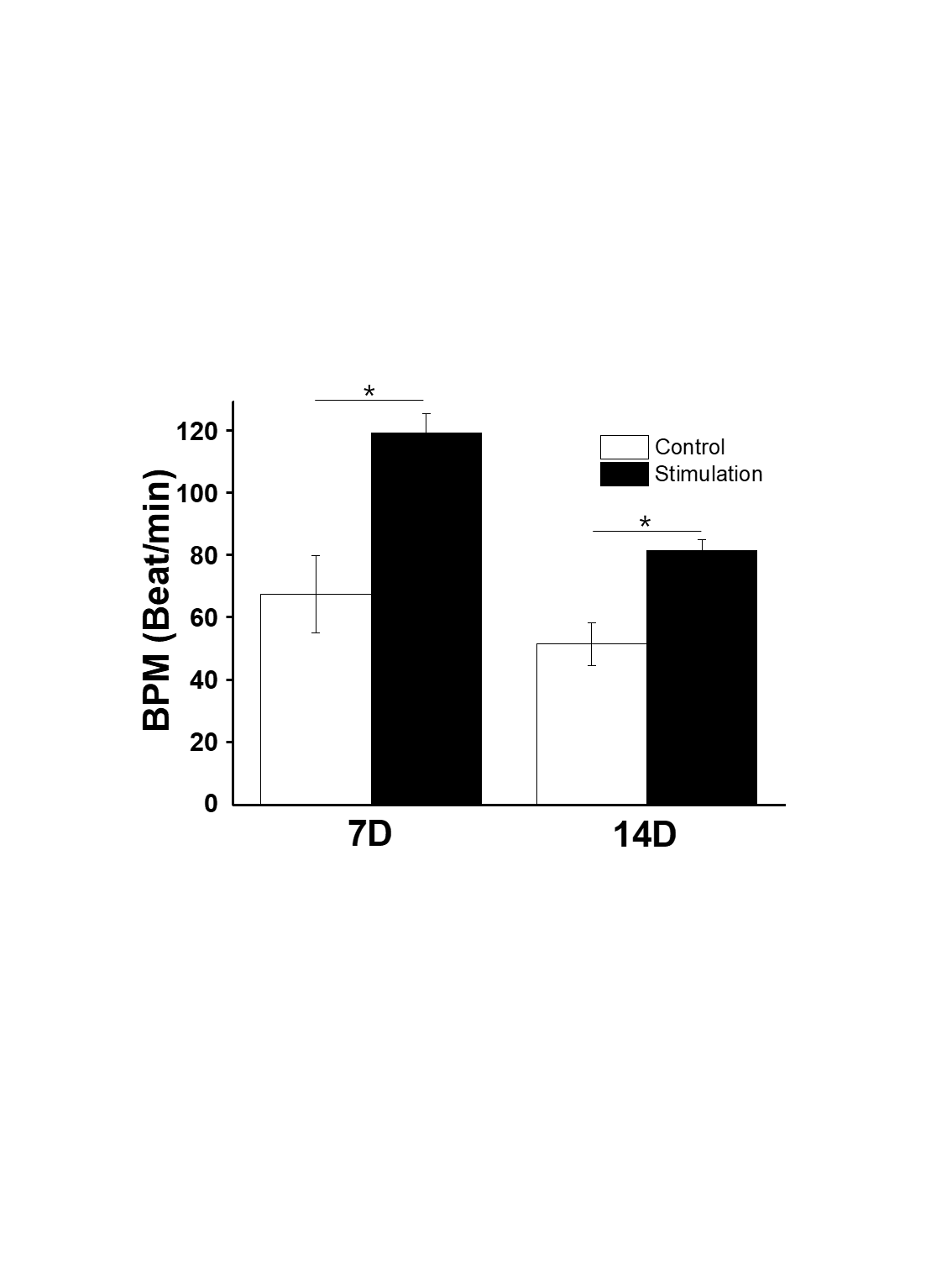

Supplement: Supplementary file 3 — Additional file 3. [file 40824_2020_189_MOESM3_ESM.tif]
